# Does Epstein-Barr virus-positive gastric cancer establish a significant relationship with the multiple genes related to gastric carcinogenesis?

**DOI:** 10.1371/journal.pone.0283366

**Published:** 2023-06-07

**Authors:** Ji Won Seo, Ki Bum Park, Hyung Min Chin, Kyong Hwa Jun

**Affiliations:** Department of Surgery, St. Vincent’s Hospital, College of Medicine, The Catholic University of Korea, Seoul, Republic of Korea; University of Nebraska-Lincoln, UNITED STATES

## Abstract

Gastric cancer has been categorized into molecular subtypes including Epstein-Barr virus (EBV)-positive tumors, which provide clinicopathological and prognostic information. In this study, we investigated the EBV infection status of patients with gastric cancer and its correlation with the clinicopathological characteristics and multiple genes related to gastric carcinogenesis. The data of 460 gastric cancer patients who underwent curative gastrectomy with D2 lymph node dissection between January 2017 and February 2022 were analyzed. The clinicopathological features and prognosis of the patients with EBV-positive gastric cancers were compared with those of EBV-negative gastric cancers. Immunohistochemistry for epidermal growth factor receptor (EGFR), C-erb B2, Ki-67, and p53 was performed. Additionally, *in situ* hybridization was conducted to detect EBV, and microsatellite instability (MSI) analysis was used to assess the deficiency in mismatch repair (MMR) genes. EBV-positivity and MSI were identified in 10.4% and 37.3% of gastric cancer patients, respectively. EBV positivity was associated with male gender (*P* = 0.001), proximal location (*P* = 0.004), poorly differentiated histological type (*P* = 0.048), moderate to severe lymphoid stroma (*P* = 0.006), high Ki-67 expression (*P* = 0.02), and a shorter resection margin. EGFR was more often expressed in EBV-negative gastric cancers (*P* < 0.001). MSI tumors were associated with older age (*P* = 0.01), the presence of lymphatic invasion (*P* = 0.02), less perineural invasion (*P* = 0.05), and the presence of *H*. *pylori* infection (*P* = 0.05). EBV positive gastric cancer is associated with increased Ki-67 and decreased EGFR expression and a shorter resection margin due to the prominent lymphoid stroma. However, MMR deficiency is not associated with EBV status even though MSI gastric cancer is related to *H*. *pylori* status.

## Introduction

Gastric cancer was the fourth most common cancer and the fifth leading cause of death due to cancer worldwide in 2020 [1]. Although the incidence and mortality of gastric cancer tended to decrease due to improved economic growth and food preservation, recurrence after surgical resection occurs in up to 40% of patients within 5 years [2,3]. A possible explanation involves the differences in the molecular phenotypes of gastric cancer [4]. Exploration for various molecular subtypes of gastric cancer has started for years. In recent, The Cancer Genome Atlas (TCGA) project has been researching gastric cancer genomics and revealed four genomic subtypes, including the Epstein-Barr virus (EBV) subtype, with increased expression of genes that suppress immunity, the microsatellite instability (MSI) subtype, with high rates of mutation, the genomically stable subtype, with less distinctive chromosomal changes, and the chromosomal instability (CIN) subtype, with marked chromosomal instability and amplified expression of receptor tyrosine kinases [5].

EBV is known to be related to various cancer types such as nasopharyngeal cancer, Hodgkin’s disease, and gastric cancer [6]. EBV-positive gastric cancer comprises about 10% of gastric cancers worldwide and is associated with proximal location, male predominance, a high incidence in the remnant stomach, and dense lymphocytic infiltration. However, there are differences in the prevalence of gastric cancer among countries [7]. EBV infection is involved in the development of atrophic gastritis, which is significantly more common in patients with *H*. *pylori* infection who take proton pump inhibitors, and the development of atrophic gastritis is a known risk factor for gastric cancer [8]. Several molecular pathways in the pathogenesis of EBV-positive gastric cancer have been elucidated [9–14], of which, abnormal methylation of multiple genes is one of the crucial initiating mechanisms in EBV-induced carcinogenesis [15].

Various genes and proteins play key roles in the development of gastric cancer. One of the most frequently mutated genes is TP53 [9]. TP53 is suppressed by mutations and loss of heterozygosity in gastric cancer. About 30–70% of gastric cancers possess TP53 point mutations [10]. Epidermal growth factor receptor (EGFR) is frequently seen during invasion and development of many malignant tumors [11]. Numerous studies have revealed that high expression of EGFR is related to gastric cancer, and 53.7% of gastric cancer tissues express EGFR [12]. The proto-oncogene C-erb B2 is one of the receptor tyrosine kinases in the C-erb B family [13]. Overexpression of C-erb B2 is widely known to be associated with breast cancer and various malignant tumors, such as ovarian, lung, and gastric carcinomas [14]. Microsatellites, which are short sequences of nucleotides, are possible to result in mutation. Microsatellite instability (MSI) is associated with numerous types of carcinomas, including gastric cancer [16]. Moreover, gastric cancer with MSI is concerned with patients with older age, less lymph node metastasis, and female [17]. To date, little is known about the association between the genes related to gastric carcinogenesis and EBV-positive gastric cancer except association of leptin-signaling-related proteins and the EBV infection status [18].

In this study, we investigated EBV infection status within gastric cancer tissues and the clinicopathological characteristics and prognosis of patients with EBV-positive cancer. We also evaluated the expression of multiple genes associated with gastric cancer and their correlation with EBV-positive gastric cancer.

## Materials and methods

### Patients and specimens

This study collected consecutively the data of 463 patients with gastric adenocarcinomas who underwent curative gastrectomy with lymphadenectomy at our institution between January 2017 and February 2022. The Institutional Review Board of St. Vincent’s Hospital, The Catholic University of Korea approved this study (VC22RISI0177). Informed consent of the patients was waived because of the retrospective nature of the study. The inclusion criteria were: (1) gastric adenocarcinoma; (2) R0 resection; (3) formalin-fixed paraffin-embedded tissue (FFPE) blocks available for analysis. Patients who had undergone previous surgery, palliative resection, chemotherapy, or radiation therapy were excluded. All patients underwent gastrectomy with lymphadenectomy based on the Korean Practice Guideline for Gastric Cancer 2018 [19]. After the surgery, clinical follow-up data were collected every 6 months during the first year and then every year thereafter. Disease-free survival (DFS) was defined as the length of time from curative surgery to the first tumor recurrence or distant metastasis. Overall survival (OS) was defined as the time from curative surgery to death from any cause.

All tissue samples were fixed in 10% buffered formalin and embedded in paraffin. Hematoxylin and eosin (H&E) stained slides, pathological reports, and other medical records were reviewed to confirm the diagnosis and the clinicopathological parameters including age, sex, American Society of Anesthesiologists (ASA) classification, tumor size, tumor location, histological type, depth of invasion, lymph node metastasis, TNM stage, lymphovascular invasion, *H*. *pylori* infection, and patient survival.

### Immunohistochemical (IHC) staining

The paraffin specimens were made into 3–5 μm sections. For immunohistochemical staining, paraffin sections were deparaffinized in xylene and rehydrated in a graded series of ethanol. After blocking peroxidase activity with hydrogen peroxide and antigen retrieval by heat induced using citrate buffer (pH 9.0), the sections were incubated at 4°C with primary antibodies against EGFR (1:500, clone 3C6, Ventana Medical Systems Inc., Tucson, AZ, USA), C-erb B2 (1:500, A0485, Dako Cytomation, Glostrup, Denmark), Ki-67 (1:100, MIB-1, Dako Cytomation, Glostrup, Denmark), and p53 (1:100, clone DO-7, Dako Cytomation, Glostrup, Denmark). Immunostaining was conducted using the ImmPRESS (MP7410-50, Vector, Burlingame, CA, USA) system and the diaminobenzidine (DAB) kit (Sk4100, Vector). After the reactions, the sections were counterstained with Meyer’s hematoxylin, dehydrated, cleared, and mounted.

### Immunohistochemical evaluation

The stained sections were scored on carcinoma cells and tumor infiltrating immune cells. Ki-67 was scored low/intermediate if ≤60% of the nuclei of cells were positive and high if >60% of the nuclei of the cells were positive. EGFR and p53 positivity were defined as brownish-yellow granular precipitation in the cell membranes and cytoplasm. The markers were scored in four categories: no staining (0), weak staining (1, <10% positive cells), intermediate staining (2, between 1 and 3), and strong staining (3, ≥50% positive cells). C-erbB2 positivity was defined by membranous staining but with no staining in cytosol. When 25% of the cells in the gastric mucous membrane were positive, the result was considered to be positive. The number of positive cells was determined by counting 100 cells within five areas.

### EBV In Situ Hybridization (ISH)

EBV status was evaluated using the Inform EBER Probe for detecting Epstein-Barr encoded RNA1 (EBER1) (800–2842, Ventana Medical Systems, Roche Diagnostics GmbH, Mannheim Germany), using the avidin-biotin peroxidase method. Samples with dark-blue staining in gastric adenocarcinoma cell nuclei were considered positive. Known EBV-positive pharyngeal tumor specimens were used as positive controls, and slides treated without the probe were used as negative controls.

### MSI analysis

To determine the MSI status of the gastric cancer specimens, MSI analysis was performed using five different mono- and dinucleotide microsatellite markers. The two mononucleotide markers consisted of BAT-25 and BAT-26. The three dinucleotide markers included D2S123, D5S346, and D17S250. The five MSI markers were divided into groups of five and co-amplified in three reaction tubes. Allelic sizes to match the tumor and normal samples were compared and considered to be MSI unstable if there was a shift of 3 bp or more in the tumor allele [20]. All tumors with one or more unstable markers were regarded as carrying some degree of instability and were defined as MSI and MSS (Microsatellite stability) when there were no unstable markers. The cutoff for classification was applied on the basis of the threshold of about 40%, which is commonly used to discriminate MSH-H and MSH-L tumors [21].

### Statistical analysis

Data were analyzed using SPSS software (ver. 21.0; SPSS Inc., Chicago, IL, USA). Continuous variables are expressed as means ± standard deviation (SD). Analysis of unpaired continuous variables was conducted by using Student’s *t*-test. Categorical variables were compared with the chi-square test. The relationship between two variables was analyzed using Pearson’s correlation analysis. Survival curves were generated by the Kaplan–Meier method and analyzed with the log-rank test. Multivariate Cox regression multivariate models were used to identify independent prognostic factors. A *P*-value < 0.05 was considered statistically significant, and all tests were two-sided unless otherwise indicated.

## Results and discussion

### Patient characteristics

The data of 460 patients were investigated in this study. Of these, the mean age was 65.4 years (SD ± 12.2, 25–92) and 69.9% of the participants were male. According to the American Society of Anesthesiologists (ASA) classification, 429 (92.7%) patients were ASA II and 34 (7.3%) patients were ASA III. Subtotal gastrectomy was performed in 404 (87.2%) patients and 240 (51.8%) patients had undifferentiated adenocarcinoma. The mean number of lymph nodes (LNs) retrieved was 43.2 (SD ± 17.1), and 161 (34.8%) patients had LN metastasis. The TNM stage was I in 285 cases (61.5%) and II/III/IV in 178 cases (38.5%). Adjuvant chemotherapy was administered to 174 (37.5%) patients. The overall 30-day mortality was 0.6% (3 patients).

ISH, IHC or the MSI analysis could not be performed for EBV, EGFR, p53, C-erb B2 and MSI in 3, 28, 37, 172, and 5 patients, respectively.

### Clinicopathological characteristics of gastric cancer according to EBV status

Of the 460 patients assessed by ISH, 48 (10.4%) had EBV-positive gastric cancer. The clinicopathological features of gastric cancer according to EBV status are summarized in Table 1. Male gender (*P* = 0.001), proximal location (*P* = 0.004), poorly differentiated histological type (*P* = 0.048), moderate to severe lymphoid stroma (*P* = 0.006) and high Ki-67 expression (*P* = 0.02) were associated with EBV-positive gastric cancer. The proximal and distal resection margins were significantly shorter in patients with EBV-positive gastric cancer (*P* = 0.02 and *P* < 0.001, respectively).

**Table 1:** Correlation between EBV infection and clinicopathological factors.

Clinicopathological factors	EBV positive, n = 48	EBV negative, n = 412	*P* value
Age (years) ≥60 <60	31 (64.6) 17 (35.4)	281 (68.2) 131 (31.8)	0.61
Sex Male Female	43 (89.6) 5 (10.4)	280 (67.9) 132 (31.1)	0.001
Tumor size (cm)	4.03±2.59	4.33±3.22	0.53
No. of tumor Single Multiple	44 (91.7) 4 (8.3)	395 (95.9) 17 (4.1)	0.18
Tumor location Upper Middle Lower Others	10 (20.8) 26 (54.2) 11 (22.9) 1 (2.1)	44 (10.7) 154 (37.4) 204 (49.5) 10 (2.4)	0.004
Histologic type Differentiated Undifferentiated	17 (35.4) 31 (64.6)	208 (50.5) 204 (49.5)	0.04
Lauren classification Diffuse Intestinal Mixed	13 (27.1) 17 (35.4) 16 (33.3)	120 (29.1) 180 (43.7) 87 (21.1)	0.27
Depth of invasion T1/T2 T3/T4	25 (52.1) 23 (47.9)	228 (55.3) 184 (44.7)	0.66
Lymph node metastasis Negative Positive	30 (68.1) 15 (31.9)	273 (66.3) 139 (33.7)	0.60
Lymphatic invasion Absent Present	32 (68.1) 15 (31.9)	230 (58.2) 165 (41.8)	0.19
Venous invasion Absent Present	43 (91.5) 4 (8.5)	325 (82.3) 70 (17.7)	0.11
Perineural invasion Absent Present	38 (80.0) 9 (20.0)	311 (79.1) 82 (20.9)	0.78
Lymphoid stroma None or mild Moderate Severe	14 (29.2) 20 (43.4) 12 (26.1)	157 (38.3) 156 (38.1) 69 (16.9)	0.006
TNM stage I II/III/IV	29 (60.4) 19 (39.6)	259 (63) 150 (37)	0.71
*H*. *pylori* infection Negative Positive	21 (58.3) 15 (41.7)	165 (52.4) 150 (47.6)	0.77
Ki-67 (%) High Low	25 (96.2) 1 (3.8)	181 (71.5) 72 (28.5)	0.02
MMR MSS MSI	30 (62.5) 18 (37.5)	236 (57.6) 174 (42.4)	0.51
Resection margin Proximal (cm) Distal (cm)	4.52±2.98 6.87±4.95	5.62±3.28 11.14±5.38	0.02 <0.001

Parentheses are percentage.

### Associations between clinicopathological features and immunohistochemical findings

The overall clinicopathological features of the patients and the immunohistochemical staining results are summarized in Table 2. Cancer progression related proteins were commonly expressed in gastric cancers; EGFR-positive in 75.6% (329/435) of gastric adenocarcinomas, p53 positive in 78.6% (335/426), and C-erb B2 positive in 8.9% (26/291). The expression of EGFR was more often positive in older age, intestinal type, and EBV-negative gastric cancers (*P* = 0.02, *P* = 0.01, and *P* < 0.001, respectively). Expression of C-erb B2 was correlated with younger age, multiple tumor masses, and advanced TNM stage (*P* = 0.05, *P* = 0.03, and *P* = 0.03, respectively).

**Table 2:** Overview clinicopathological features and immunohistochemical staining results.

Clinicopathological factors	EGFR		*P* value	p53		*P* value	C-erb B2			*P* value
	Negative	Positive		Negative	Positive		Negative	Positive		
Age (years)	62.71±11.66	65.85±12.36	0.02	64.67±10.95	65.23±12.66	0.69	64.08±11.77	68.77±11.83	0.05
Sex Male Female	77 29	231 98	0.46	67 (73.6) 24 (26.4)	234 (69.9) 101 (30.1)	0.48	192 (72.5) 73 (27.5)	19 (73.1) 7 (26.9)	0.94
Tumor size (cm)	3.95±3.05	4.54±3.24	0.09	4.41±3.22	4.39±3.25	0.94	4.27 ±3.44	4.67 ±2.74	0.56
No. of tumor Single Multiple	102 4	85 14	0.66	87 (95.6) 4 (4.4)	322 (96.1) 13 (3.9)	0.82	257 (97.0) 8 (3.0)	23 (88.5) 3 (11.5)	0.03
Tumor location Upper Middle Lower Others	14 38 50 4	36 132 154 7	0.54	13 (14.3) 32 (35.2) 43 (47.3) 3 (3.3)	35 (10.4) 136 (40.6) 156 (46.6) 8 (2.4)	0.63	21 (12.1) 107 (40.4) 119 (44.9) 7 (2.6)	3 (11.5) 7 (26.9) 14 (53.8) 2 (22.2)	0.32
Histologic type Differentiated Undifferentiated	46 (43.4) 59 (55.7)	163 (49.5) 163 (49.5)	0.54	51 (56.0) 39 (42.9)	156 (46.6) 176 (52.5)	0.26	123 (48.3) 133 (50.2)	16 (61.5) 10 (38.5)	0.59
Lauren classification Diffuse Intestinal Mixed	33 (31.1) 40 (37.7) 21 (19.8)	94 (28.6) 145 (44.1) 78 (23.7)	0.01	23 (25.3) 43 (47.3) 20 (22.0)	101 (30.1) 140 (41.8) 76 (22.7)	0.77	71 (26.8) 114 (43) 65 (24.5)	4 (15.4) 17 (65.4) 5 (19.2)	0.13
Depth of invasion T1/T2 T3/T4	75 (70.8) 31 (29.2)	216 (66.1) 111 (33.9)	0.37	64 (70.3) 26 (28.6)	222 (66.3) 112 (33.4)	0.43	185 (69.8) 78 (29.4)	16 (61.5) 10 (38.5)	0.58
Lymph node metastasis Negative Positive	71 (67.0) 35 (38.0)	208 (54.7) 121 (36.8)	0.48	54 (59.3) 37 (40.7)	219 (65.4) 116 (34.6)	0.28	181 (68.3) 84 (31.7)	15 (57.7) 11 (42.3)	0.27
Lymphatic invasion Absent Present	62 (58.5) 37 (34.9)	180 (54.7) 140 (42.6)	0.09	48 (52.7) 39 (42.9)	192 (57.3) 131 (39.1)	0.72	161 (60.8) 93 (35.1)	12 (46.2) 14 (53.8)	0.12
Venous invasion Absent Present	85 (80.2) 14 (13.2)	260 (79.0) 60 (18.2)	0.10	72 (79.1) 15 (16.5)	266 (79.4) 57 (17.0)	0.93	211 (79.6) 43 (16.2)	19 (73.1) 7 (26.9)	0.24
Perineural invasion Absent Present	79 (74.5) 19 (17.9)	248 (75.4) 71 (21.6)	0.10	65 (71.4) 22 (24.2)	256 (76.4) 65 (19.4)	0.59	198 (74.7) 54 (20.4)	20 (76.9) 6 (23.1)	0.50
TNM stage I II/III/IV	72 (67.9) 37 (32.1)	195 (59.3) 134 (40.7)	0.43	56 (61.5) 35 (38.5)	206 (61.5) 129 (38.5)	0.99	177 (66.8) 88 (33.2)	12 (46.2) 14 (53.8)	0.03
*H*. *pylori* infection Negative Positive	35 (33.0) 46 (43.4)	139 (42.2) 109 (33.1)	0.12	38 (41.8) 36 (39.6)	133 (39.7) 114 (34.0)	0.30	112 (42.3) 101 (38.1)	12 (46.2) 9 (34.6)	0.92
EBV infection Negative Positive	82 (77.4) 24 (22.6)	310 (94.2) 19 (5.8)	<0.001	85 (93.4) 6 (6.6)	299 (89.3) 36 (10.7)	0.23	237 (89.4) 25 (10.6)	25 (96.2) 1 (2.6)	0.27

Parentheses are percentage.

### Clinicopathological characteristics according to MMR deficiency

Among the 458 gastric cancers, 37.3% (171/458) had MSI. The remaining 287 (62.7%) patients were classified as MSS. The clinicopathological features of gastric cancers according to MMR deficiency are summarized in Table 3. Older age (*P* = 0.01), presence of lymphatic invasion (*P* = 0.02), less perineural invasion (*P* = 0.05), and the presence of *H*. *pylori* infection (*P* = 0.05) were related to MMR deficiency. MMR deficiency was not correlated with EBV infection status (*P* = 0.98).

**Table 3:** Overview clinicopathological features and MSI analysis.

Clinicopathological factors	MMR		*P* value
	MSS	MSI	
Age (years)	63.9±11.75	66.9±12.72	0.01
Sex Male Female	207 (72.1) 80 (27.9)	114 (66.7) 57 (33.3)	0.21
Tumor size (cm)	4.26±3.44	4.37±2.74	0.71
No. of tumor Single Multiple	276 (96.2) 11 (3.8)	161 (94.2) 10 (5.8)	0.31
Tumor location Upper Middle Lower Others	35 (12.2) 113 (39.4) 130 (45.3) 9 (3.1)	18 (10.5) 67 (39.2) 84 (49.1) 2 (1.2)	0.50
Histologic type Differentiated Undifferentiated	142 (49.5) 141 (49.1)	77 (45.0) 93 (54.4)	0.59
Lauren classification Diffuse Intestinal Mixed	74 (25.8) 128 (44.6) 70 (24.4)	59 (34.5) 67 (39.2) 33 (33.9)	0.14
Depth of invasion T1/T2 T3/T4	199 (69.3) 86 (30.0)	113 (66.1) 58 (33.9)	0.38
Lymph node metastasis Negative Positive	195 (67.9) 92 (32.1)	106 (62.0) 65 (38.0)	0.19
Lymphatic invasion Absent Present	163 (63.4) 94 (36.6)	97 (53.0) 86 (47.0)	0.02
Venous invasion Absent Present	227 (79.1) 49 (17.1)	139 (81.3) 25 (14.6)	0.78
Perineural invasion Absent Present	194 (76.1) 61 (23.9)	153 (83.6) 30 (16.4)	0.05
TNM stage I II/III/IV	172 (64.2) 96 (35.8)	115 (60.5) 75 (39.5)	0.42
*H*. *pylori* infection Negative Positive	121 (42.2) 109 (38.0)	56 (32.7) 64 (37.4)	0.05
EBV infection Negative Positive	238 (88.8) 30 (11.2)	153 (90.5) 18 (9.5)	0.55

Parentheses are percentage.

### Prognosis and survival analysis

**Fig 1:**
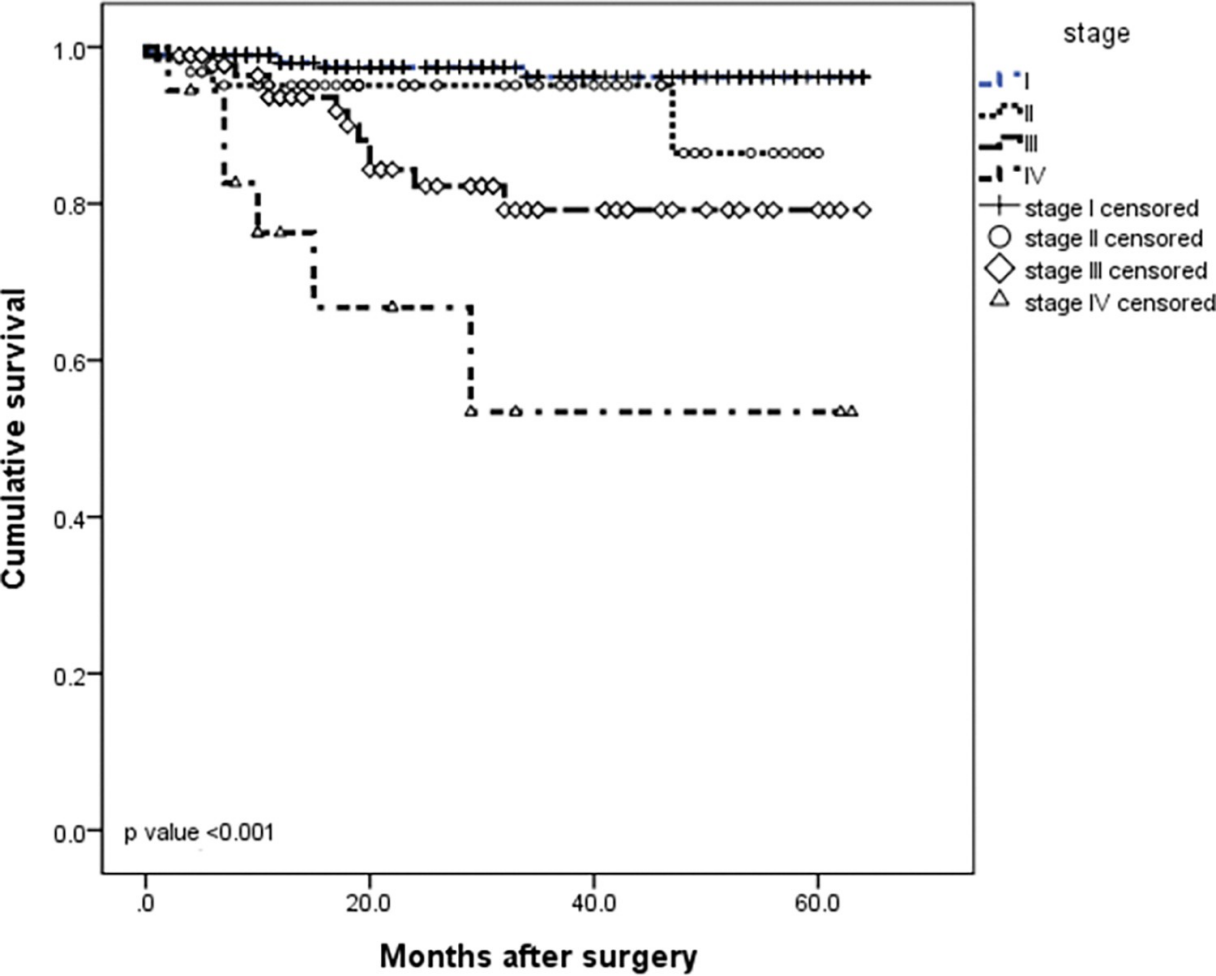
Kaplan–Meier survival curve analysis. Advanced TNM stage was associated with poor overall survival (OS).) in patients with gastric adenocarcinoma (*P* < 0.001 and *P* < 0.001, respectively).

**Fig 2:**
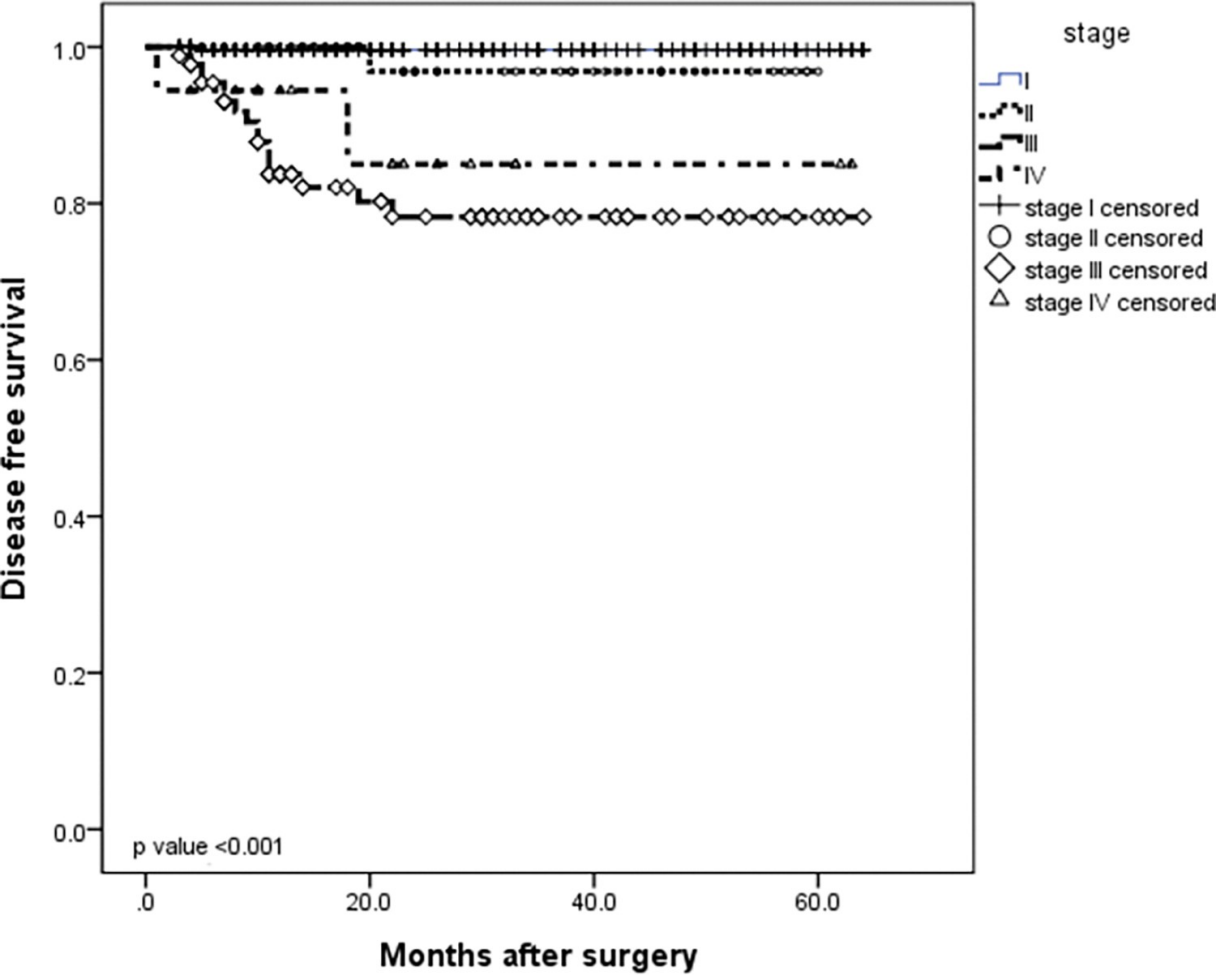
Meier survival curve analysis. **Kaplan–** Advanced TNM stage was associated with poor disease-free survival (DFS) in patients with gastric adenocarcinoma (*P* < 0.001 and *P* < 0.001, respectively).

**Fig 3:**
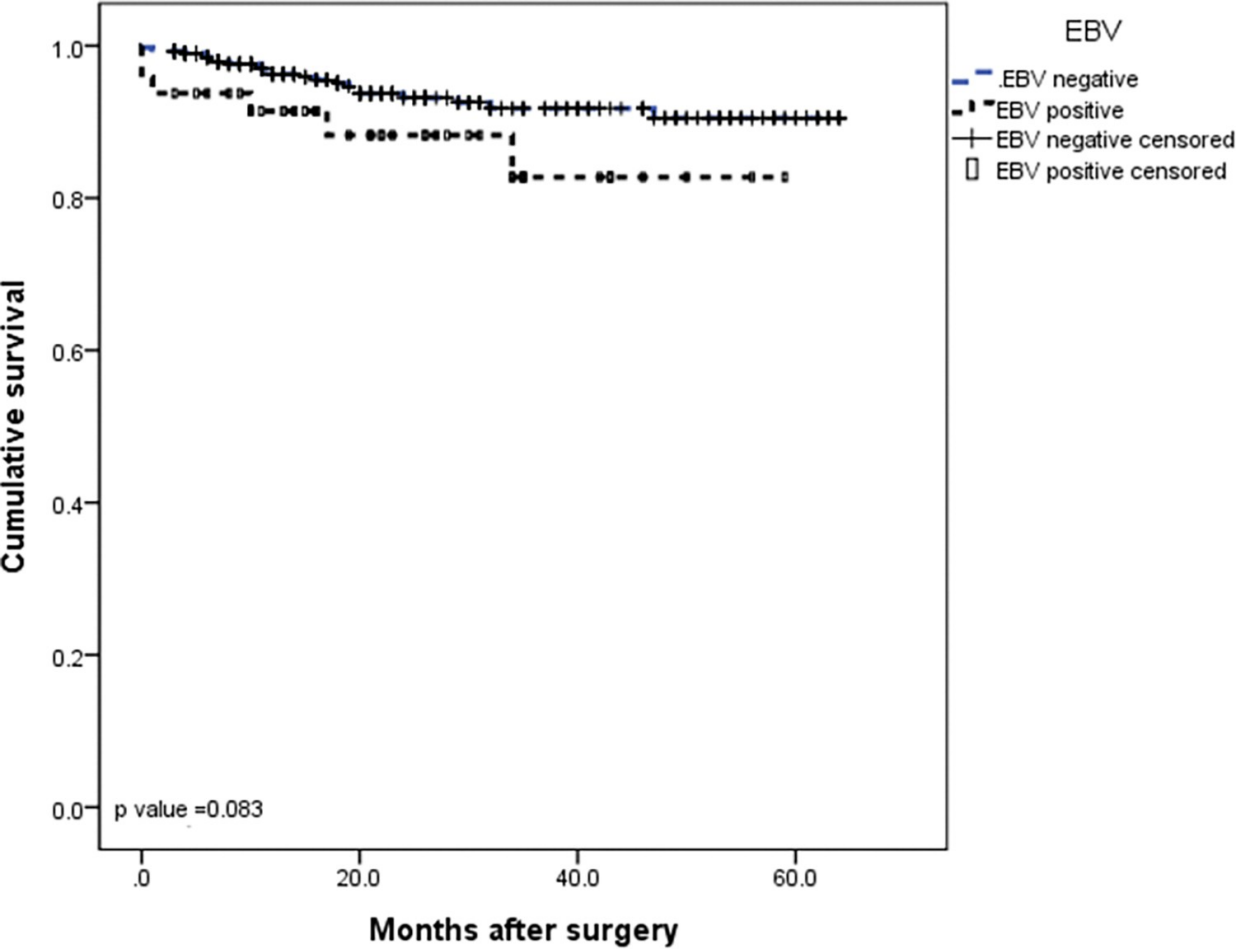
Kaplan-Meier survival curve analysis. OS of gastric cancer patients according to EBV status.

**Fig 4:**
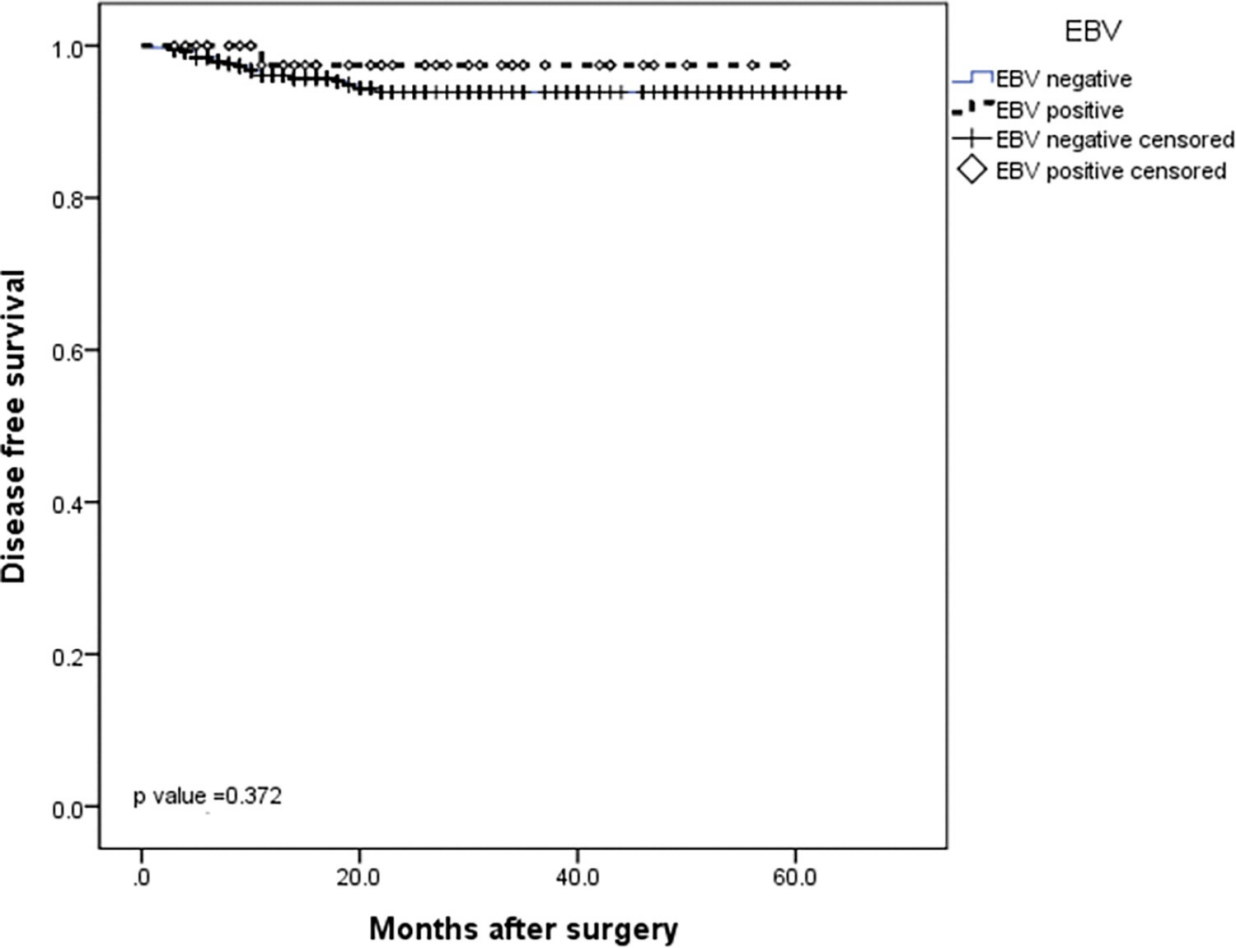
Kaplan-Meier survival curve analysis. DFS of gastric cancer patients according to EBV status.

**Fig 5:**
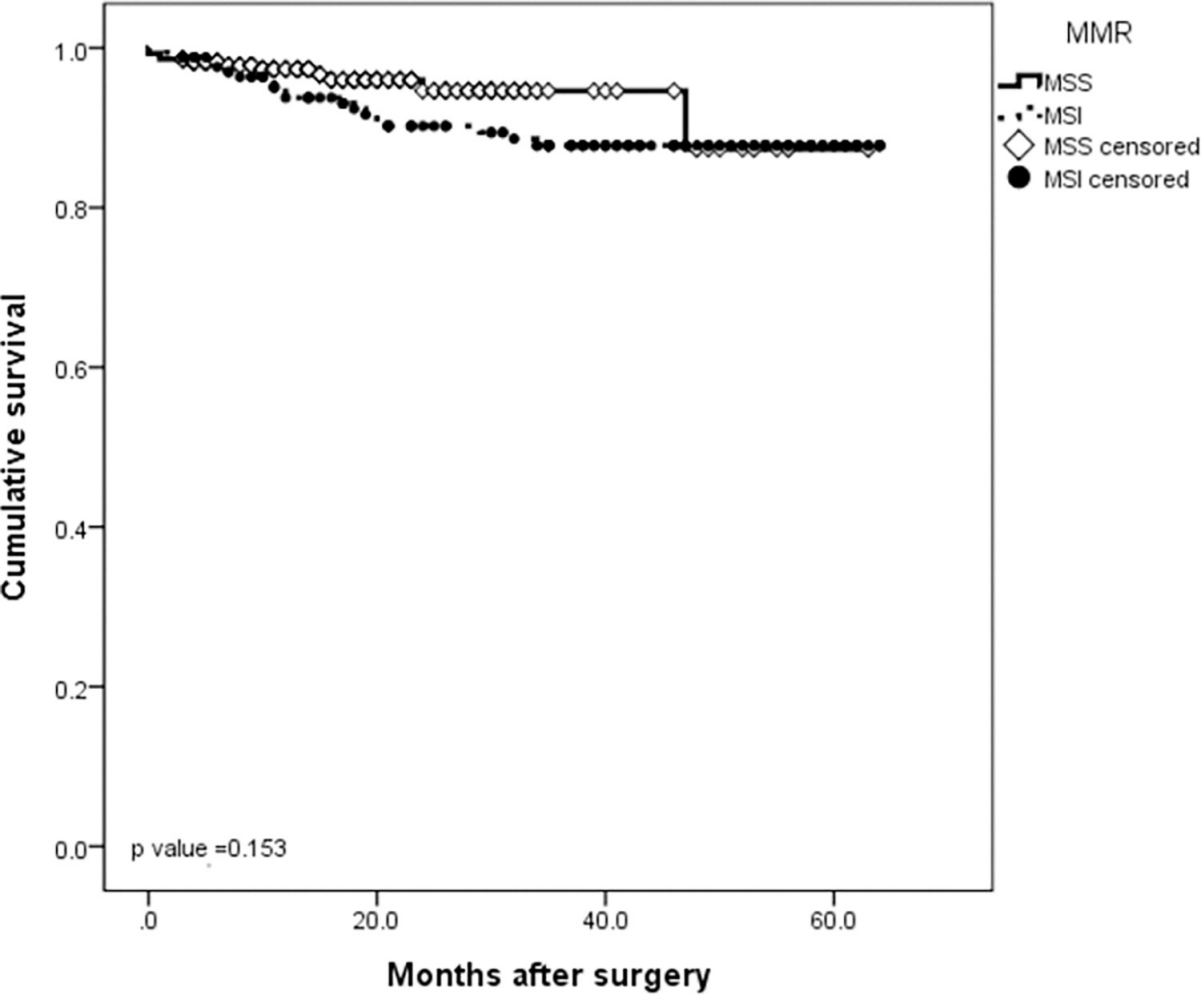
Kaplan-Meier survival curve analysis. OS of gastric cancer patients according to MSI status.

**Fig 6:**
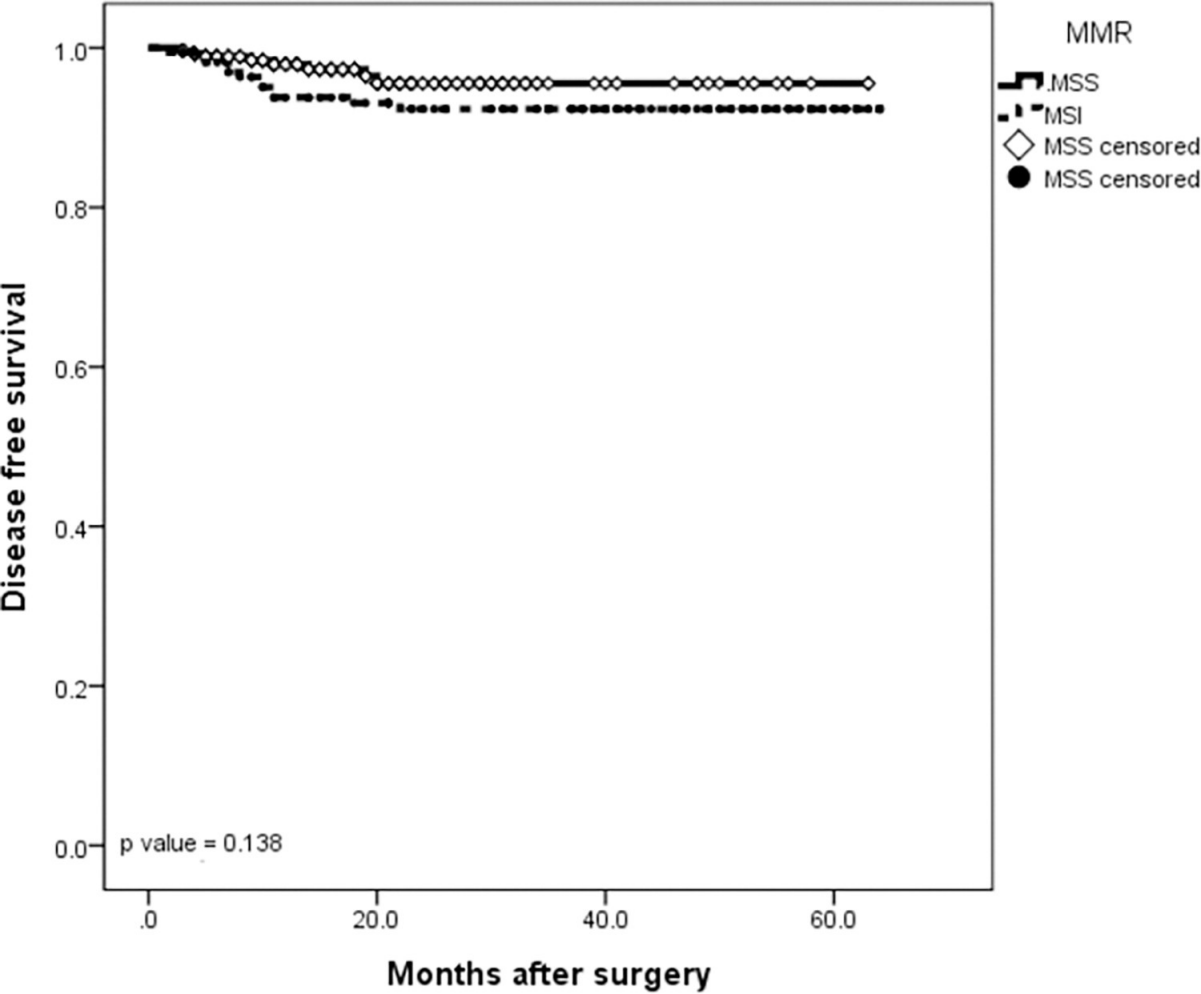
Kaplan-Meier survival curve analysis. DFS of gastric cancer patients according to MSI status.

The median follow-up was 24.5 months (IQR 3–64 months). At the time of this study, twenty patients had disease recurrence (6 locoregional and 14 distant) and 29 patients died. The 5-year DFS and 5-year OS rates for the entire cohort were 95.7% and 93.7%, respectively (Figs 1 and 2). When evaluating survival considering only EBV status, no significant difference was observed between EBV-negative and EBV-positive patients in the DFS rate (93.9% *vs* 97.4%, respectively, *P* = 0.37) or in the 5-year OS rate (90.5% *vs* 82.7%, respectively, *P* = 0.08) (Figs 3 and 4). Similarly, considering only MSI status, the 5-year DFS rate was superior in the MSS group (95.5% *vs* 92.3%, *P* = 0.13), but the result was not statistically significant. The MSI patients tended to have slightly longer 5-year OS compared to MSS patients (87.3% *vs* 87.8%, *P* = 0.15), but the result was not statistically significant (Figs 5 and 6).

Univariate and multivariate analyses were performed to evaluate the prognostic factors affecting 5-year DFS and 5-year OS (Table 4). Univariate analysis revealed that undifferentiated histological type (*P* = 0.003), positive LN metastasis (*P* < 0.001), advanced TNM stage (*P* < 0.001), lymphatic invasion (*P* = 0.002), and venous invasion (*P* < 0.001) were associated with poor OS. Also, undifferentiated histologic type (*P* = 0.03), positive LN metastasis (*P* < 0.001), advanced the TNM stage (*P* < 0.001), lymphatic invasion (*P* < 0.001), and venous invasion (*P* < 0.001) were associated with poor DFS. However, multivariate analysis identified only TNM stage as an independent factor associated with poorer 5-year OS and DFS (*P* = 0.05 and *P* = 0.02, respectively). Furthermore, the presence of EBV and MSI status were not significant prognostic factors for survival or recurrence in the Cox regression multivariate analysis.

**Table 4:** Survival analysis of variables predicting the risk of death and recurrence with gastric cancers.

Variables	Significance univariate	Significance multivariate	Hazard ratio	95% CI
Overall survival				
Age (<60 years *vs* ≥60 years) Sex (male *vs* female) Histologic type (undifferentiated *vs* differentiated) LN metastasis (positive *vs* negative) TNM stage (I *vs* II, III, IV) Lymphatic invasion (absent *vs* present) Venous invasion (absent *vs* present) EBV infection (negative *vs* positive) MMR (MSI *vs* MSS)	0.47 0.63 0.003 <0.001 <0.001 0.002 <0.001 0.08 0.15	0.23 0.58 0.11 0.43 0.05 0.11 0.09 0.15 0.35	1.65 1.25 0.45 0.64 3.30 0.49 2.08 2.00 1.45	0.723–3.762 0.552–2.870 0.172–1.222 0.218–1.917 0.989–11.056 0.203–1.197 0.874–4.961 0.778–5.181 0.640–3.179
Disease-free survival				
Age (<60 years *vs* ≥60 years) Sex (male *vs* female) Histologic type (undifferentiated *vs* differentiated) LN metastasis (positive *vs* negative) TNM stage (I *vs* II, III, IV) Lymphatic invasion (absent *vs* present) Venous invasion (absent *vs* present) EBV infection (negative *vs* positive) MMR (MSI *vs* MSS)	0.81 0.30 0.03 <0.001 <0.001 <0.001 <0.001 0.37 0.13	0.65 0.26 0.32 0.44 0.02 0.85 0.68 0.19 0.19	1.27 0.52 0.57 0.56 22.65 1.09 1.19 0.26 1.81	0.469–3.326 0.168–1.630 0.194–1.713 0.131–2.455 1.600–320.883 0.434–2.811 0.495–2.906 0.033–2.027 0.734–4.491

## Discussion

In the present study, 10% of gastric cancer patients were EBV-positive, and male gender, proximal location, poorly differentiated histological type, and moderate to severe lymphoid stroma were associated, as previous studies have investigated. Moreover, high Ki-67 expression and low expression of EGFR expression have been demonstrated to be associated with EBV-positive gastric cancer. Several studies have indicated that EBV-positive gastric cancer has a better prognosis than EBV-negative gastric cancer [22]. Nevertheless, in this study, no significant differences in the 5-year DFS and OS were observed. A plausible explanation for this result is that mortality of EBV-positive gastric cancer patients’ is mostly related to their comorbidities. Only 2.1% of EBV-positive patients died of gastric cancer recurrence. On the other hand, in EBV-negative group, cancer recurrence comprised 64% of total mortalities.

EBV-positive gastric cancer was associated with moderate to severe lymphoid stroma according to our results. Gastric cancer with lymphoid stroma is defined as undifferentiated cancer cells encircled by lymphoid stroma with reactive lymphoid follicles [23]. EBV participates in the establishment of gastric cancer with lymphoid stroma, which is also called lymphoepithelioma-like carcinoma [24]. The relationship between EBV-positive gastric cancer and the lymphoid stroma may contribute to the submucosal spread of tumor cells, even though a definite mechanism has not been verified. The lymphoid stroma has an expansive pattern of growth throughout the tumor [25]. In this study, the proximal and distal margins of the EBV-positive group were significantly shorter than those of the EBV-negative group. Prominent lymphoid infiltration could affect the extension of cancer cells into the submucosal layer, but it needs to be elucidated.

In this study, strong Ki-67 expression was concerned with EBV-positive gastric cancer. Twenty-nine EBV-positive patients (96.2%) expressed high levels of Ki-67. Ki-67 is widely known as a significant tumor proliferation marker [26]. A meta-analysis reported that high Ki-67 expression is not associated with lymph node metastasis or tumor stage, but it may be a marker for poor prognosis in gastric cancer patients [27]. However, the role of Ki-67 as a clinicopathological factor in gastric cancer is vague [28]. No study has investigated the relationship between Ki-67 expression and EBV-positive gastric cancer. The molecular mechanism of high Ki-67 expression and EBV infection is unknown. It is plausible that EBV infection status affects active cell proliferation, which is associated with Ki-67 expression. Future investigation is necessary to determine how these two factors influence each other.

High EGFR expression is closely associated with gastric cancer. According to the present study, high EGFR expression was related to older age, intestinal type in the Lauren classification, and EBV-negative gastric cancer. Although the oncogenic role of EGFR is widely known, there is no agreement on the association between EGFR expression and the prognosis of gastric cancer [29]. Moreover, even though the clinicopathological mechanism for the correlation between EGFR and gastric cancer is unclear, some studies have reported that it is related to older age, moderately to poorly differentiated histology, and a higher stage cancer [30,31]. To the best of our knowledge, connection of EGFR status and EBV infection has not been revealed. This study determined that high EGFR expression status was significantly associated with EBV-negative gastric cancer. It could be that high EGFR expression status prevents EBV infection, even though the molecular mechanism is indistinct.

MSI is characterized by various sizes of repetitive sequences and is caused by a failure of the DNA mismatch repair (MMR) gene [32]. In this study, 10% of patients were EBV-positive and 39.6% had MSI, which is comparable to the rates seen in the TCGA (9% and 22% of gastric cancer, respectively) [5]. Moreover, EBV-positive and MSI tumors are distinct clinicopathological entities. Similar to previous literatures, EBV-positive gastric cancer was associated with male patients and a proximal location, an undifferentiated histological type, and prominent lymphoid stroma [33]. In contrast, MSI in gastric cancer was associated with lymphatic invasion, less perineural invasion, and *H*. *pylori* infection. Additionally, MSI gastric cancer occurred more frequently in older patients than MSS gastric cancer, as epigenetic changes including methylation intensify with age [34]. A previous study described that EBER expression and MMR status are not associated [35]. Although EBV infection status and MMR deficiency are significant molecular subtypes of gastric cancer, both factors do not affect each other. That is, they are mutually exclusive.

Gastric cancer is one of the few malignant diseases known to play a role in the etiology of infectious agents such as *H*. *pylori* and EBV. It has been suggested that EBV and *H*. *pylori* co-infection synergistically cause an increase in gastric cancer [36,37]. Several studies have suggested that *H*. *pylori* and EBV coinfection may be synergistically more effective in gastric carcinogenesis by playing an active role in modulating the transformation of EBV from latent to lytic phase in gastric tissues [38,39]. In this study, the prevalence of *H*. *pylori* and EBV co-infection was 41.7% of gastric cancer cases even though the correlation was not statistically significant. Souza *et al*. reported that *H*. *pylori* and EBV co-infection were detected in 1.85% (1/54) of juvenile gastritis patients, 5.4% (2/37) of adult gastritis patients, and 9.6% (12/125) of gastric cancer patients [40]. They found that *H*. *pylori* alone was responsible for 78.4% of these cases, but no statistically significant difference was found in the *H*. *pylori* alone cases, *H*. *pylori* and EBV co-infection could not have had a synergistic effect on the formation of gastric cancer. The etiological role of *H*. *pylori* in EBV-positive gastric cancer needs to be elucidated in future study.

This study had several limitations. First, the sample size was relatively small, which may have caused different outcomes from previous studies. According to several studies, EBV-positive gastric cancer is associated with less lymph node metastasis and a better prognosis. However, in this study, no significant relationship was detected between EBV positive gastric cancer and lymph node metastasis or prognosis. This outcome may be related to the small sample size. Second, this was a retrospective single-center-study. Therefore, selection bias may have affected the outcome. Moreover, multicenter studies are necessary for validity. Finally, immunohistochemistry inhomogeneity was found in this study. Applying immunohistochemistry to all gastric cancer patients who had undergone surgery was difficult in this center. The incompleteness of the immunohistochemistry might have led to insignificant outcomes in this study.

## Conclusions

EBV-positive gastric cancer is associated with increased Ki-67 and decreased EGFR expression and shorter resection margins due to prominent lymphoid stroma. However, MMR deficiency is not associated with EBV status even though MSI gastric cancer is associated with *H*. *pylori* status. EBV positivity might be an additional criterion for the margin resection in both gastrectomized and endoscopic resected specimens.

## Supporting information

S1 Data (XLSX)
